# Severe asthmatic airways have distinct circadian clock gene expression pattern associated with WNT signaling

**DOI:** 10.1002/clt2.12379

**Published:** 2024-06-28

**Authors:** Nguyen Quoc Vuong Tran, Minh Khang Le, Yuki Nakamura, Atsuhito Nakao

**Affiliations:** ^1^ Department of Immunology Faculty of Medicine University of Yamanashi Yamanashi Japan; ^2^ Department of Human Pathology University of Yamanashi Yamanashi Japan; ^3^ Yamanashi GLIA Center University of Yamanashi Yamanashi Japan; ^4^ Atopy Research Center Juntendo University School of Medicine Tokyo Japan

To the Editor,

The circadian clock, which consists of a network of approximately 30 clock genes, enables organisms to coordinate physiological processes including airway function in synchrony with the changing 24‐h environment.^1^ Asthma is characterized by a marked day–night variation in symptoms and laboratory parameters in the airways, suggesting that the airway circadian clock underpins the pathology of asthma.^2^, ^3^ However, the basic question, “do asthmatic airways have normal or altered circadian clock activity?” remains unanswered. This study analyzed the expression profiles of circadian clock genes and their potential significance in asthmatic airways using a public database of patients with asthma.

Gene expression data from bronchial epithelial brushing samples of patients with mild/moderate and severe asthma and healthy subjects were obtained from five publicly available NCBI‐GEO datasets (GSE41861, GSE43696, GSE63142, GSE67472, and GSE89809). Two bronchial epithelial brushing sample datasets of COPD patients (GSE20257 and GSE37147) and two peripheral blood sample datasets of patients with asthma (GSE69683 and GSE207751) were used as controls. Information of datasets analyzed in this study was summarized in Table S1.

Differential gene expression analysis of 34 circadian clock genes showed that *NR1D2*, *PER2*, and *PER3* are downregulated in bronchial epithelial samples from patients with asthma, apparently in those from severe asthma in four of the five datasets (4/5) compared with normal subjects (Figure 1A,B, Figure S1A, Table S2). Considering the lack of information on the timing of sampling, we conducted a sensitivity analysis for gene expression using relative amplitude data for *NR1D2*, *PER2*, and *PER3* from CircaDB, a database of circadian gene expression profile.^4^ Even after accounting for diurnal variations, the differences in *PER2* expression remained significant in 3/5 datasets, while for *NR1D2* and *PER3*, significance was observed in only 2/5 datasets (Figure 1C, Figure S1B). Corroborating our findings, a previous study using time‐matched bronchial brushing samples showed that the expression of *NR1D2* and *PER2* was reduced in asthma patients, as determined by Real‐time PCR.^5^

**FIGURE 1:**
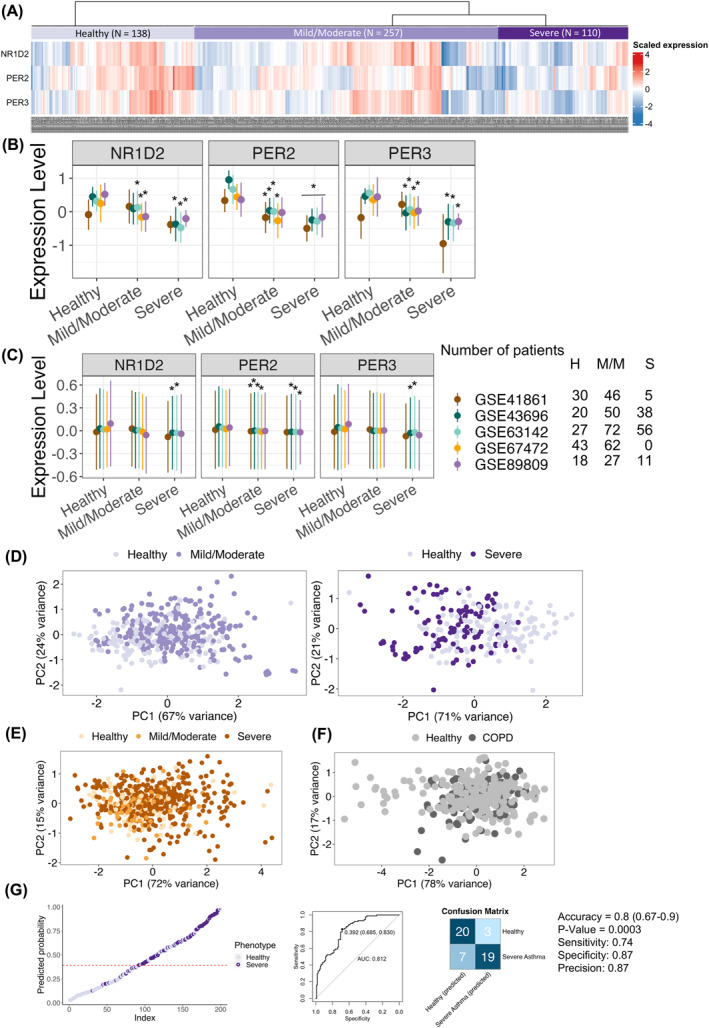
Altered expression of *NR1D2*, *PER2*, and *PER3* in severe, but not mild/moderate, asthmatic airways. (A) Heatmap of *NR1D2*, *PER2*, *PER3* expression across healthy, mild/moderate, and severe asthma groups. (B) The expression levels of *NR1D2*, *PER2*, *PER3* were compared between healthy subjects and asthma patients across five datasets. (C) Sensitivity analysis for *NR1D2*, *PER2*, *PER3* across datasets, using relative amplitude from CircaDB to estimate daily oscillations. H, healthy; M/M mild/moderate; S, severe. To facilitate visualization in panels (B and C), the expression level of each gene was scaled within each dataset. Median and IQR are presented, analyzed via Kruskal–Wallis one‐way ANOVA, statistical significance was adopted from Figure S1A,B, respectively. (D) PCA reveal distinct expression patterns of *NR1D2*, *PER2*, *PER3* between healthy and severe asthma patients (right panel), not evident in mild/moderate cases (left panel). (E) PCA of *NR1D2*, *PER2*, *PER3* in asthma blood samples could not differentiate patient groups. (F) PCA of *NR1D2*, *PER2*, *PER3* in COPD bronchial brushing samples could not distinguish healthy subjects from COPD patients. (G) Logistic regression model based on *NR1D2*, *PER2*, *PER3* expressions discriminates healthy from severe asthma patients. For analysis, 248 subjects were split into a training set (*n* = 199) and a testing set (*n* = 49). Left panel: training set predictions (ROC cut‐off at 0.392); right panel: confusion matrix and accuracy for the testing set.

Importantly, in bronchial epithelial tissue, dimension reduction by principal component analysis and t‐distributed stochastic neighbor embedding using the expression of *NR1D2*, *PER2*, and *PER3* showed distinct clustering between healthy subjects and patients with severe asthma (Figure 1D, Figure S1C). For comparison, we performed the same exploration on two peripheral blood sample datasets of patients with asthma and two bronchial epithelial brushing sample datasets of COPD patients. The distinct cluster was not seen in blood sample analyses from asthma patients despite the significantly downregulation of NR1D2, PER2, and PER3 (GSE69683, GSE207751) (Figure 1E, Figure S2) or in COPD (GSE20257, GSE37147) (Figure 1F, Figure S3). Thus, the alterations in *NR1D2*, *PER2*, and *PER3* may be specific to the severe asthmatic airways. Furthermore, a logistic regression model using these three genes successfully distinguishing healthy subjects from patients with severe asthma with high accuracy (0.8, 95% CI: 0.66–0.9, AUC: 0.81) (Figure 1G).

To explore possible pathophysiological significance of the alterations in three clock gene expression in severe asthmatic airways, we identified 41 genes correlated with *NR1D2*, *PER2*, or *PER3* that were differentially expressed in healthy subjects and patients with severe asthma (Table S3 and S4). Network analysis with STRING identified interactions between these circadian genes and a node centered around Catenin Beta 1 (*CTNNB1*), a key component of WNT signaling pathway (Figure 2A). Pathway analysis showed significant reduced activity of WNT and also NOTCH signaling pathways in severe asthma in 3/4 applicable datasets (Figure 2B).

**FIGURE 2:**
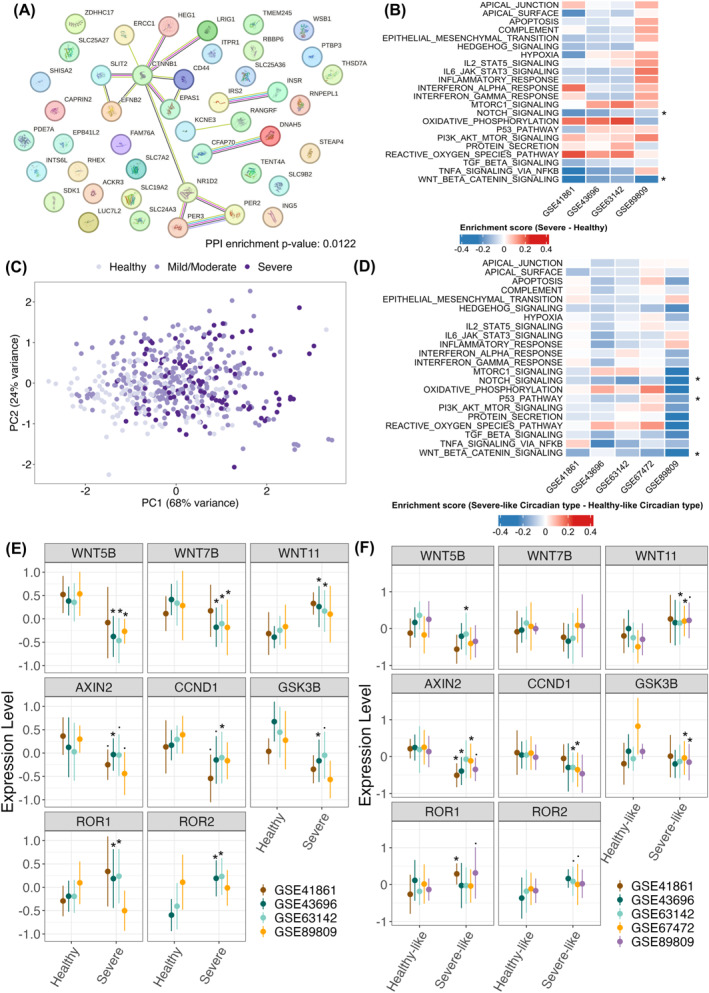
Altered expression of *NR1D2*, *PER2*, and *PER3* is associated with decreased activity of WNT signaling in severe or severe‐like asthmatic airways. (A) Protein‐Protein Interaction (PPI) network analysis of NR1D2, PER2, PER3, and 41 related genes performed with STRING database. (B) Heatmap of pathway analysis enrichment score differences between healthy subjects and severe asthma patients in four datasets. (C) PCA analyses of NR1D2, PER2, and PER3 expressions suggest that mild/moderate asthma patients may exhibit either healthy or severe asthma circadian types. (D) Heatmap of pathway analysis enrichment score differences between healthy‐like and severe‐like circadian types in five datasets. *p ≤ 0.05 in at least three datasets. (F) The expression levels of WNT signaling related genes (*WNT5B*, *WNT7B*, *WNT11, AXIN2, CCND1, GSK3B, ROR1,* and *ROR2*) were compared between healthy subjects and severe asthma patients across four applicable datasets. (G) The expression levels of WNT signaling related genes were compared between healthy‐like and severe‐like circadian type across five datasets. To facilitate visualization in panels (E and F), the expression level of each gene was scaled within each dataset. Statistical significance was adopted from Figures S4 and S5, respectively.

Patients with mild/moderate asthma were interspersed among both healthy controls and severe asthma patients in the dimension reduction analysis (Figure 2C), suggesting that their circadian gene expression patterns may align with either the healthy‐like or severe‐like circadian type. To explore this further, the logistic regression model (Figure 1E) was used to categorize patients with mild/moderate asthma into either the healthy‐like or severe‐like circadian type. Pathway analysis showed significant reduced WNT and also NOTCH signaling activity in patients with the severe‐like circadian type in 3/5 datasets (Figure 2D) as well as in severe asthma patients (Figure 2B).

Further analysis revealed changes in WNT signaling genes in severe asthma: *WNT5B* and *WNT7B* were downregulated, while *WNT11* was upregulated. Downstream, genes of canonical WNT signaling (*AXIN2*, *CCND1*, *GSK3B*) trended toward downregulation, whereas non‐canonical signaling genes (*ROR1*, *ROR2*) showed potential upregulation (Figure 2E, Figure S4). Interestingly, this pattern, especially *WNT11* upregulation and canonical signaling genes downregulation, was also seen in the severe‐like circadian phenotype (Figure 2F, Figure S5). WNT11 is known to induce non‐canonical WNT signaling and contributed to airway remodeling in asthma.^6^, ^7^ Conversely, canonical WNT signaling is reported to protect against allergic airway disease.^8^ Collectively, these findings strongly support the idea that altered expression of *NR1D2*, *PER2*, and *PER3* in severe asthmatic airways is associated with altered activity of WNT and NOTCH signaling possibly via *CTNNB1*. Recently, the interaction between circadian clock and WNT signaling plays important role in lung regeneration,^9^ which may be important for airway remodeling in asthma. Future research using single‐cell RNA sequencing of asthma airways could reveal the complex relationships between the circadian clock, WNT signaling, and asthma pathology.

This study has several limitations. Firstly, the lack of information on specific sampling times restricted our analysis although bronchial epithelial brushing samples are likely collected during regular operation hours and we have considered diurnal variations in our analysis (Figure 1C). Secondly, we cannot exclude potential influence of asthma treatment including corticosteroid,^10^ since most patients in the datasets, except for GSE67472, were on asthma medication. Thirdly, the causal link between the altered expression of these genes and reduced WNT signaling activity in severe asthma remains uncertain. Considering the bidirectional interaction of the circadian clock with key signaling pathways, these factors might jointly contribute to severe asthma development.^11^ Further experimental and clinical studies is necessary to overcome these limitations.

In summary, we show that severe asthmatic airways have distinct circadian clock gene expression patterns associated with WNT signaling, a crucial pathway for airway remodeling in asthma. Thus, disturbance of airway circadian clock activity may be one of the determining features of severe asthma.

## AUTHOR CONTRIBUTIONS

**Nguyen Quoc Vuong Tran**: Conceptualization; data curation; formal analysis; writing—original draft. **Minh Khang Le**: Data curation; formal analysis; writing—original draft. **Yuki Nakamura**: Formal analysis; writing—original draft; **Atsuhito Nakao**: Conceptualization; formal analysis; funding acquisition; writing—original draft.

## CONFLICT OF INTEREST STATEMENT

The authors declare no conflicts of interest.

## Supporting information

Supplementary Material S1

Table S1

Table S2

Table S3

Table S4
