# Supplementary material for: Severe asthmatic airways have distinct circadian clock gene expression pattern associated with WNT signaling
Source: Clin Transl Allergy. 2024 Jun 28;14(7):e12379. doi: 10.1002/clt2.12379 (PMC11213687; doi:10.1002/clt2.12379)
Supplement: Supplementary file 1 — Supplementary Material S1 [file CLT2-14-e12379-s004.docx]

**Supplementary Materials and Methods**

**Materials**

All study data are publicly available from NCBI-GEO database. GEO accession for bronchial epithelial brushing samples of asthma: GSE41861, GSE43696, GSE63142, GSE67472, and GSE89809; for peripheral blood samples of asthma: GSE69683 and GSE207751; and for bronchial epithelial brushing samples of COPD GSE20257 and GSE37147. Detailed information for the datasets included is provided in Table S1.

**Circadian clock genes expression analysis**

Thirty-three circadian clock genes including in the KEGGs Homo sapiens Circadian rhythm pathway (<https://www.genome.jp/entry/pathway+hsa04710>) are included for analysis. Because the platform, the baseline expression, and batch effect in each dataset was different, we chose to compare the expressions of circadian clock gene in healthy subjects, patients with mild/moderate asthma, and patient with severe asthma in each dataset separately. Genes that were consistently up/downregulated in all datasets and reach statistical significance in at least four out of five datasets were selected for further analysis, including NR1D2, PER2, and PER3. For visualization, the expressions of these genes were scaled using robust data scaling in each dataset to make consistent with the usage of median and interquartile range (IQR) in single comparison. This approach involves adjusting each variable by subtracting the median and dividing by the interquartile range (IQR), the difference between the 75th and 25th percentiles. The formula for scaling is: scaled_value = (value - median) / (75th percentile - 25th percentile).

**Sensitivity analysis**

We extracted the relative amplitude (rAMP) data for NR1D2, PER2, and PER3 in the human lung from CircaDB ^1^. The respective rAMP values for NR1D2, PER2, and PER3 in the human lung were found to be 0.541399, 0.482806, and 0.710504. The rAMP is calculated by dividing the differences in daily expression by the baseline expression. In our analysis, we treated each sample's expression as the baseline expression. The maximum expression for each gene was then determined by adding the product of the baseline expression and rAMP to the baseline expression. Conversely, the minimum expression was obtained by subtracting this product from the baseline expression. Consequently, for each gene, we acquired three distinct values per subject: maximum expression, baseline, and minimum expression. We compiled these values to conduct a comprehensive gene expression analysis, aiming to ascertain the persistence of statistical significance.

**Dimension reduction**

After scaling, the five datasets were combined, comprising a total of 505 subjects (138 healthy, 257 with mild/moderate asthma, and 110 with severe asthma).

Dimension reduction was conducted using principal component analysis (PCA) and t-Distributed Stochastic Neighbor Embedding (t-SNE). This workflow was also applied for dimension reduction in two datasets of peripheral blood samples from asthma patients and two datasets of bronchial epithelial brushing samples from COPD patients.

**Pathway analysis**

Pathway analysis was performed using the refined hallmark pathways provided by The Molecular Signatures Database (MSigDB) ^2,3^. The Hallmark pathways were utilized to reduce redundancy and to focus on pathways particularly relevant to asthma ^2^. Following the Practical Guidelines for Using the MSigDB Hallmark Gene Set Collection as recommended by the authors, we also double-checked the founder gene sets extracted from the C2:CP:Reactome in The Molecular Signatures Database (MsigDB 7.0). Gene set variation analysis (GSVA) ^4^ was performed. We then conducted ANOVA and used the Benjamini-Hochberg correction method to compare the enrichment score of each pathway between each group and to minimize the false positive results, respectively.

To construct heatmap summarizing pathway analysis results, the differences between the two groups were calculated and are displayed as color ranging from blue to red as shown in the key of the heatmap.

**Analysis platform**

All analysis was performed using R version 4.3.2 ^6^ and Bioconductor version 3.18 ^7^.

**Reference:**

1. Pizarro A, Hayer K, Lahens NF, Hogenesch JB. CircaDB: a database of mammalian circadian gene expression profiles. *Nucleic Acids Res*. Jan 2013;41(Database issue):D1009-13. doi:10.1093/nar/gks1161

2. Liberzon A, Birger C, Thorvaldsdottir H, Ghandi M, Mesirov JP, Tamayo P. The Molecular Signatures Database (MSigDB) hallmark gene set collection. *Cell Syst*. Dec 23 2015;1(6):417-425. doi:10.1016/j.cels.2015.12.004

3. Subramanian A, Tamayo P, Mootha VK, et al. Gene set enrichment analysis: a knowledge-based approach for interpreting genome-wide expression profiles. *Proc Natl Acad Sci U S A*. Oct 25 2005;102(43):15545-50. doi:10.1073/pnas.0506580102

4. Hanzelmann S, Castelo R, Guinney J. GSVA: gene set variation analysis for microarray and RNA-seq data. *BMC Bioinformatics*. Jan 16 2013;14:7. doi:10.1186/1471-2105-14-7

5. Szklarczyk D, Franceschini A, Wyder S, et al. STRING v10: protein-protein interaction networks, integrated over the tree of life. *Nucleic Acids Res*. Jan 2015;43(Database issue):D447-52. doi:10.1093/nar/gku1003

6. *_R: A Language and Environment for Statistical Computing_*. R Foundation for Statistical Computing; 2023. <<https://www.R-project.org/>>.

7. Huber W, Carey VJ, Gentleman R, et al. Orchestrating high-throughput genomic analysis with Bioconductor. *Nat Methods*. Feb 2015;12(2):115-21. doi:10.1038/nmeth.3252

**Supplementary Figures**


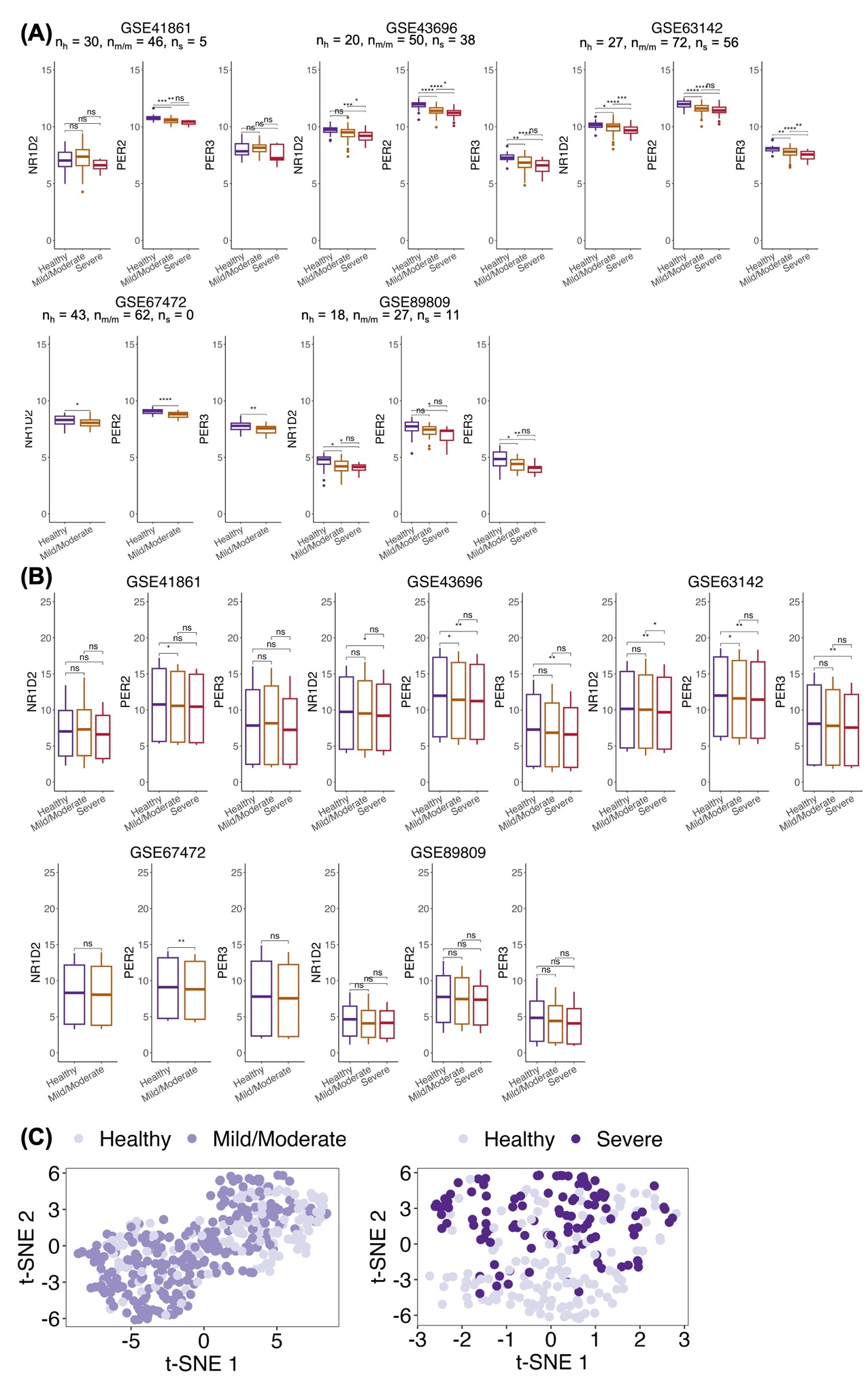


Figure S1. Expression levels of *NR1D2*, *PER2*, and *PER3* in healthy subjects and patients with asthma among five datasets.

(A) Comparative expression of *NR1D2*, *PER2*, *PER3* across five datasets in healthy individuals and asthma patients. n_h_, n_m/m_, and n_s_ denote numbers of healthy, mild/moderate, and severe asthma subjects, respectively.

(B) Sensitivity analysis of *NR1D2*, *PER2*, *PER3* across five datasets, considering daily oscillations estimated using relative amplitude from CircaDB.

(C)t-SNE for healthy subjects and patients with mild/moderate asthma (left panel) or patients with severe asthma (right panel)

All boxplots show median and IQR, analyzed via Kruskal-Wallis one-way ANOVA. Significance levels: *p<0.05, **p<0.01, ***p<0.001, ****p<0.0001, ns = nonsignificant.


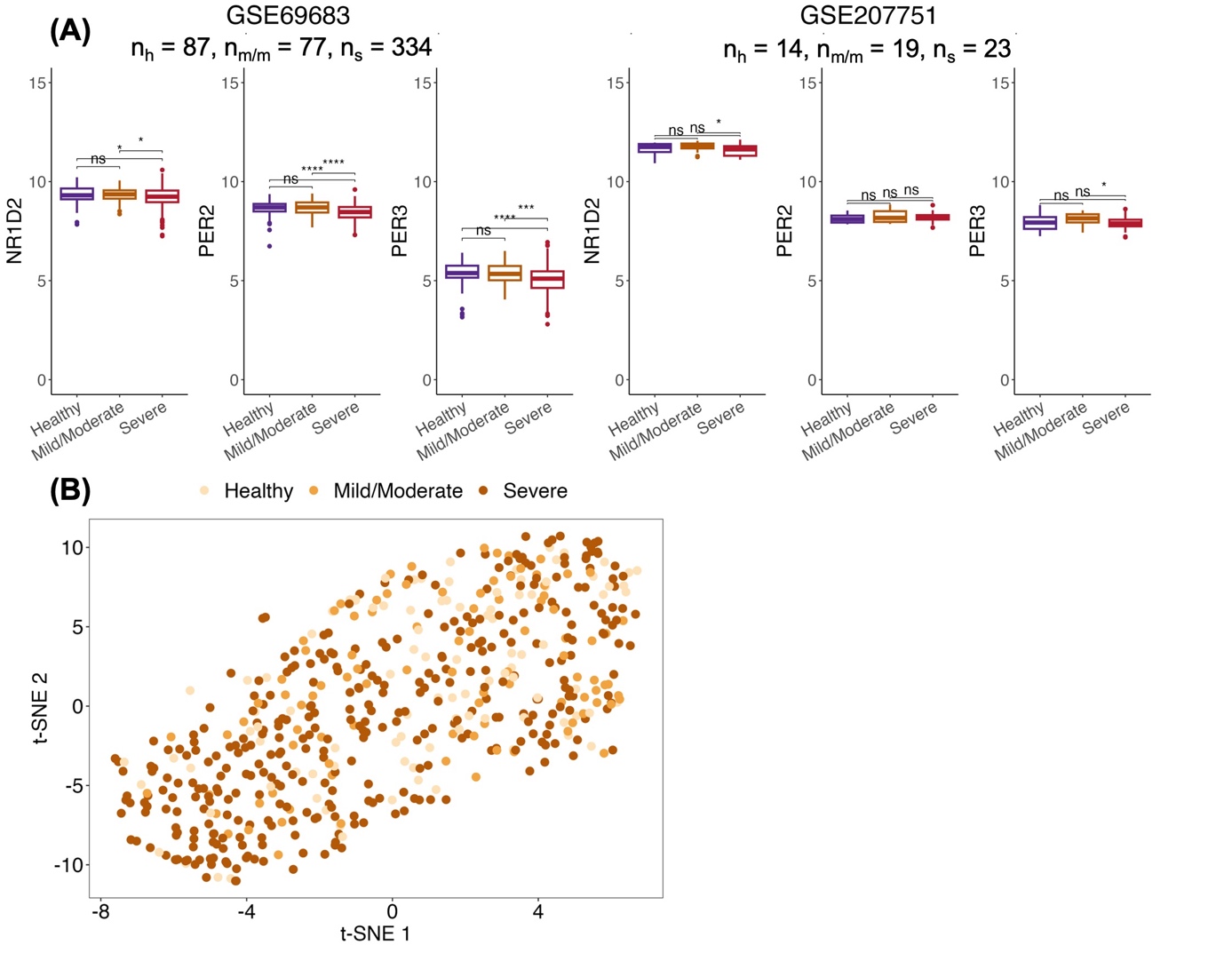


Figure S2. Differential expression and associated pathways of *NR1D2*, *PER2*, and *PER3* in the blood samples of asthma patients using GSE69683 and GSE207751.

(A) Boxplot represents expression levels of *NR1D2*, *PER2*, and *PER3* in healthy subjects and patients with asthma. n_h_, n_m/m_, and n_s_ denote numbers of healthy, mild/moderate, and severe asthma subjects, respectively. Median and IQR are shown, analyzed via Kruskal-Wallis one-way ANOVA. *p<0.05, **p<0.01, ***p<0.001, ****p<0.0001, ns = nonsignificant.

(B) Dimension reduction by t-SNE using the expression of *NR1D2*, *PER2*, and *PER3* in blood samples could not separate healthy subjects and patients with mild/moderate or severe asthma.


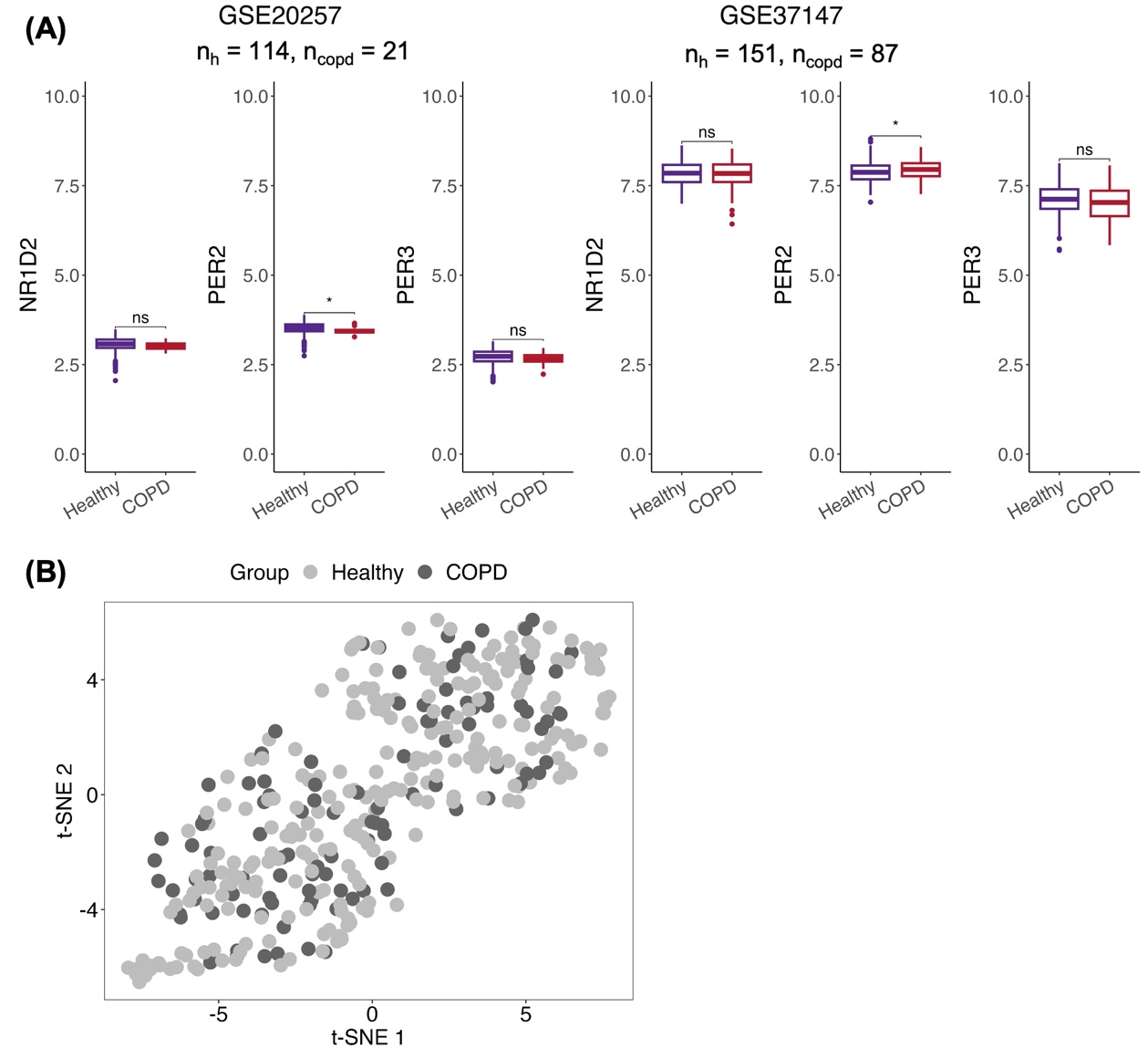
Figure S3. In two COPD bronchial brushing dataset, NR1D2 was only lower in COPD patients in GSE20257. Despite being statistically significance, the expression of PER2 were inconsistent between the two datasets.

(A). Expression levels of NR1D2, PER2, and PER3 in two COPD datasets. n_h_ and n_copd_ denote numbers of healthy and COPD subjects, respectively. Median and IQR are shown, analyzed via Kruskal-Wallis one-way ANOVA. *p<0.05, ns = nonsignificant.

(B) Dimension reduction by t-SNE using the expression of *NR1D2*, *PER2*, and *PER3* could not separate healthy subjects and COPD patients.


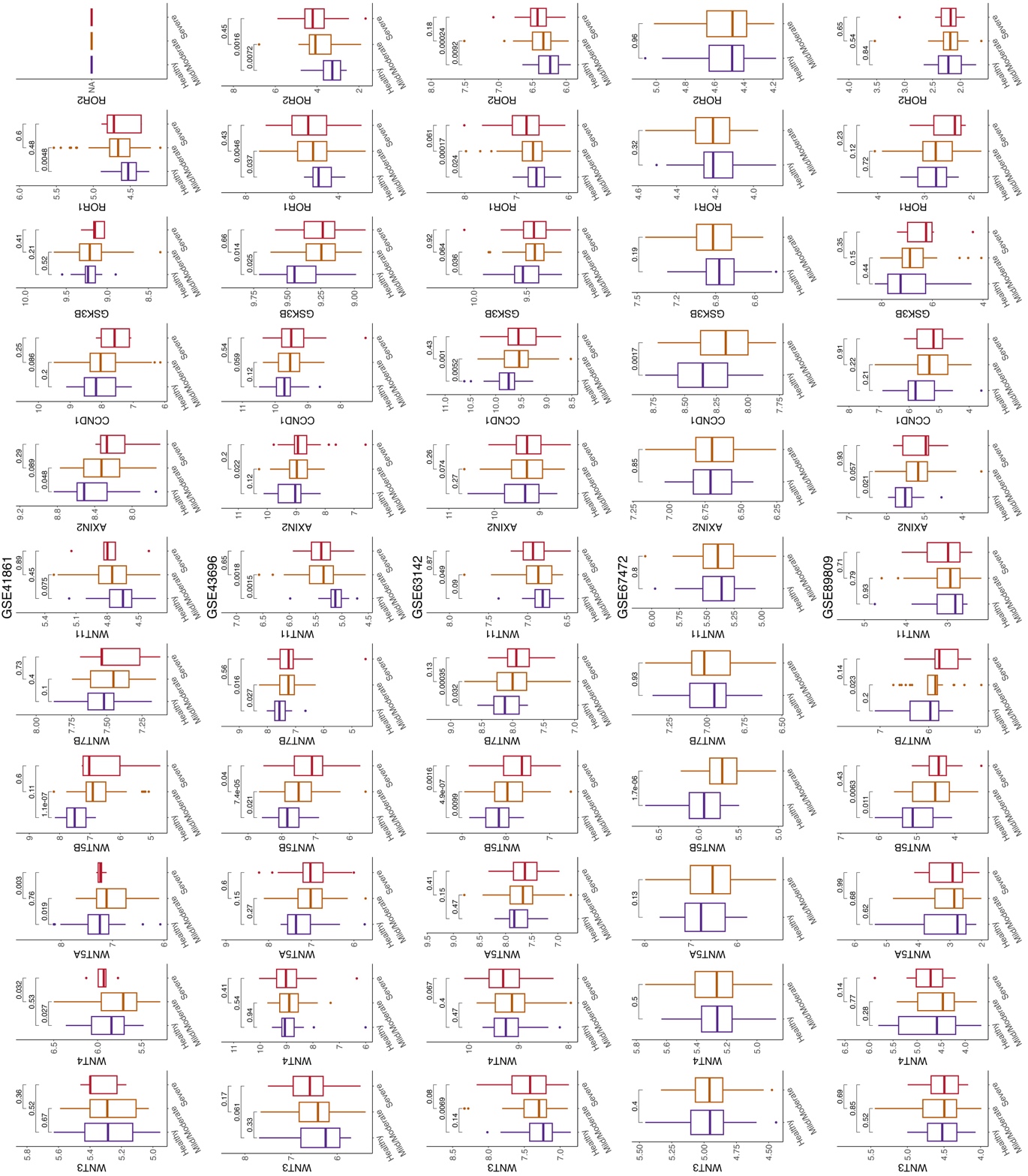
Figure S4. Expression levels of WNT family genes *(WNT3, WNT4, WNT5A, WNT5B, WNT7B,* and *WNT11)*, canonical WNT signalings genes (*AXINA2*, *CCND1*, and *GSK3B*), and non-canonical WNT signaling (*ROR1* and *ROR2*) in healthy subjects and patients with asthma among five datasets. Boxplots show median and IQR, analyzed via Kruskal-Wallis one-way ANOVA. P-value is denoted for each comparison.


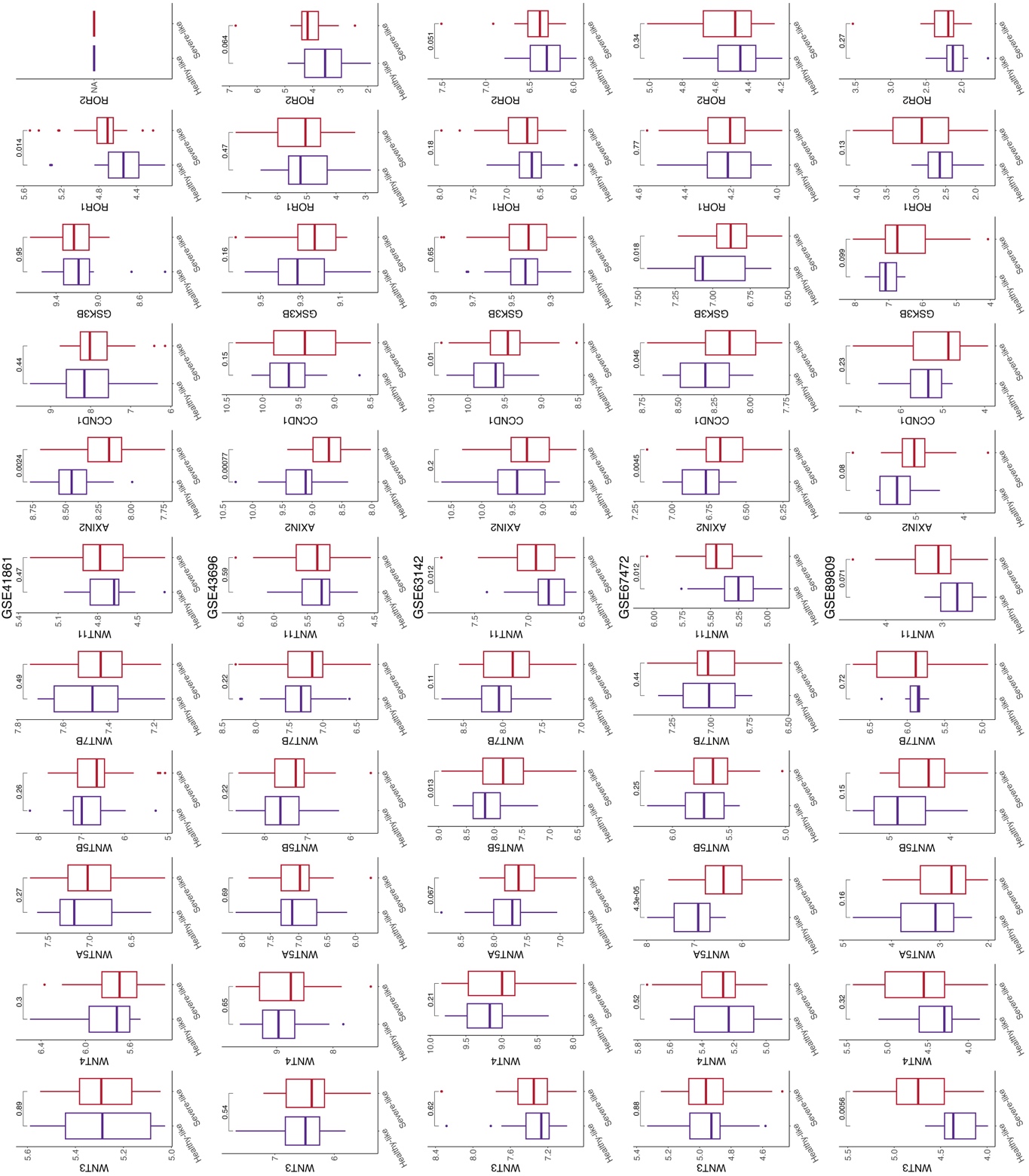
 Figure S5. Expression levels of WNT family genes *(WNT3, WNT4, WNT5A, WNT5B, WNT7B,* and *WNT11)*, canonical WNT signalings genes (*AXINA2*, *CCND1*, and *GSK3B*), and non-canonical WNT signaling (*ROR1* and *ROR2*) in Mild/moderate asthma subjects with healthy-like circadian type and severe-like circadian type among five datasets. Boxplots show median and IQR, analyzed via Kruskal-Wallis one-way ANOVA. P-value is denoted for each comparison.
